# The structure of pyogenecin immunity protein, a novel bacteriocin-like immunity protein from *Streptococcus pyogenes*

**DOI:** 10.1186/1472-6807-9-75

**Published:** 2009-12-17

**Authors:** Changsoo Chang, Penny Coggill, Alex Bateman, Robert D Finn, Marcin Cymborowski, Zbyszek Otwinowski, Wladek Minor, Lour Volkart, Andrzej Joachimiak

**Affiliations:** 1 Midwest Center for Structural Genomics and Structural Biology Center, Biosciences Division, Argonne National Laboratory, Argonne, Illinois 60439, USA; 2 Wellcome Trust Sanger Institute, Wellcome Trust Genome Campus, Hinxton, CB10 1SA, UK; 3 Midwest Center for Structural Genomics, Department of Molecular Physiology and Biological Physics, University of Virginia, Charlottesville, VA 22903, USA; 4 Midwest Center for Structural Genomics, Department of Biochemistry, UT Southwestern Medical Center at Dallas, Dallas, TX 75235, USA

## Abstract

**Background:**

Many Gram-positive lactic acid bacteria (LAB) produce anti-bacterial peptides and small proteins called bacteriocins, which enable them to compete against other bacteria in the environment. These peptides fall structurally into three different classes, I, II, III, with class IIa being pediocin-like single entities and class IIb being two-peptide bacteriocins. Self-protective cognate immunity proteins are usually co-transcribed with these toxins. Several examples of cognates for IIa have already been solved structurally. *Streptococcus pyogenes*, closely related to LAB, is one of the most common human pathogens, so knowledge of how it competes against other LAB species is likely to prove invaluable.

**Results:**

We have solved the crystal structure of the gene-product of locus Spy_2152 from *S. pyogenes*, (PDB:2fu2), and found it to comprise an anti-parallel four-helix bundle that is structurally similar to other bacteriocin immunity proteins. Sequence analyses indicate this protein to be a possible immunity protein protective against class IIa or IIb bacteriocins. However, given that *S. pyogenes *appears to lack any IIa pediocin-like proteins but does possess class IIb bacteriocins, we suggest this protein confers immunity to IIb-like peptides.

**Conclusions:**

Combined structural, genomic and proteomic analyses have allowed the identification and *in silico *characterization of a new putative immunity protein from *S. pyogenes*, possibly the first structure of an immunity protein protective against potential class IIb two-peptide bacteriocins. We have named the two pairs of putative bacteriocins found in *S. pyogenes *pyogenecin 1, 2, 3 and 4.

## Background

Many Gram-positive bacteria produce anti-bacterial peptides and small proteins, called bacteriocins. There are three main classes produced by Gram-positive lactic acid bacteria (LAB): class I bacteriocins are the lantibiotics, small (<4 kDa), post-translationally modified peptides containing unusual amino acids such as lanthionine; class II are small, unmodified, heat-stable bacteriocins (<10 kDa); class III include larger (>30 kDa) heat-labile proteins, such as murein hydrolases [1]. Most bacteriocins are synthesized as precursors, which are matured and secreted, then target a specific bacterium and kill it by increasing its membrane permeability to various small molecules. Class II bacteriocins are subdivided into IIa, pediocin-like unmodified bacteriocins, IIb, two-peptide unmodified bacteriocins, IIc, formerly class V, where the N- and C-termini are covalently linked resulting in a cyclic structure, and class IId, non-pediocin, single, linear peptides [2]. The genetics and biosynthesis of class IIa bacteriocins have been well studied [3], and these constitute one of the most important groups of antimicrobial peptides, due to their useful antibacterial properties. All known IIa bacteriocins are described as being active against *Listeria *and some have already been tested as food preservatives for controlling food-borne pathogens [4]. Structurally, class IIa bacteriocins are related to each other being unstructured in aqueous solution, but with a central amphiphilic alpha-helical region when in lipid micelles or TFE [5-7]; they contain the characteristic conserved N-terminal YGNGVxCxxxxC sequence, though usually not the GxxxG motif(s) characteristic of IIb and IIc bacteriocins [7].

The class IIb two-peptide unmodified bacteriocins, for example plantaricin E/F [8], need the complementary action of both peptides to be active [9]. These bacteriocins contain long amphiphilic alpha-helical stretches, and the two complementary peptides interact when exposed to membrane-like entities. The GxxxG motif is conserved in many two-peptide bacteriocins, and it is postulated that the two complementary peptides dimerize via a helix-helix interaction that involves this motif, to form the functionally active heterodimer [10]. The dimer functions by creating a pore within the membrane through which small molecules leak out, and, typically, the genes encoding the two peptides are found adjacent to each other on the same operon [10].

The cyclic class IIc bacteriocins are characterized by being tryptophan-rich and lacking any GG, GxxxG or YGNGVxCxxxxC motifs, [11]; the class IId bacteriocins have none of these features but some have recently been found also to be circular, rather than linear, with conserved AxxhhN and AhhW/F motifs [12-14].

In order to neutralize the toxic effect of the peptide on the "producing" cell the genes encoding bacteriocins are generally co-transcribed with a cognate immunity protein. These small proteins (typically 88-115 amino acids) interact very tightly with a specific bacteriocin or pair of bacteriocins and protect the "producing" microbe from the toxic effect of its own bacteriocin [15,16]. The immunity proteins usually show high specificity for their cognate bacteriocins [17,18]. For each class IIa bacteriocin encoded in a genome there is a one to one relationship between bacteriocin and cognate immunity protein; whereas, in contrast, for each pair of class IIb two-peptide bacteriocins there is a single cognate immunity protein encoded in a genome [9]. Structures of five immunity proteins have already been solved: ImB2 [19], EntA-im [20], PedB [21], PisI [22], and Mun-Im [23], all protective against IIa bacteriocins. As yet, no structures for immunity proteins protective against IIb or IIc bacteriocins have been solved.

The sequencing of bacterial genomes, including those from human pathogens, has revealed a number of genes which might potentially code for new bacteriocins and immunity proteins, suggesting that the use of these antimicrobial peptides is more widespread than previously thought and that bacteria might be targeting several different bacterial species using these toxins. Understanding which specific immunity protein neutralizes which bacteriocin toxin is important if these peptides are to be used as antimicrobials.

The Gram-positive bacterium *Streptococcus pyogenes*, closely related to LAB, is one of the most common human pathogens. It causes a wide range of both minor diseases such as pharyngitis, erysipelas and pyodermas, that are readily controlled by antibiotics, as well as major, often lethal, conditions such as acute rheumatic fever, necrotizing fasciitis and streptococcal toxic shock syndrome, in developing countries and in the western world [24]. The search for new antibacterial agents effective against this species is thus urgent.

Other streptococcal species have been shown to secrete bacteriocin-like toxins, as there are reports of *S. salivarius *producing a variety of bacteriocin-like inhibitory substances showing *in vitro *inhibitory activity against *S. pyogenes*, including Salivaricin A [25,26]. Such observations suggest that antibacterial toxins are playing a very important role in controlling the level of the *S. pyogenes *population in human microbiomes. Bacteriocin-like toxins and antitoxins may well have an impact on the development of new antibacterial strategies and treatments. A thorough understanding of the biology of the bacteriocins in combination with their immunity proteins is important for any possible therapeutic use of bacteriocins. The full sequences of LAB genomes provide opportunities to scan for the presence of toxins and their corresponding immunity proteins. However, the sequence alone may not be sufficient to identify these proteins.

Here we present the first structure of the protein from locus Spy_2152 (gene names taken from *S. pyogenes *M1 GAS), named pyogenecin immunity protein Sp-PIP, determined at 2.15 Å resolution. We provide structural and sequence analyses, and identify the putative corresponding bacteriocin-like toxins in the *S. pyogenes *genome, which are found to belong to the class IIb two-peptide bacteriocins.

## Results

### Structure determination

**Figure 1:**
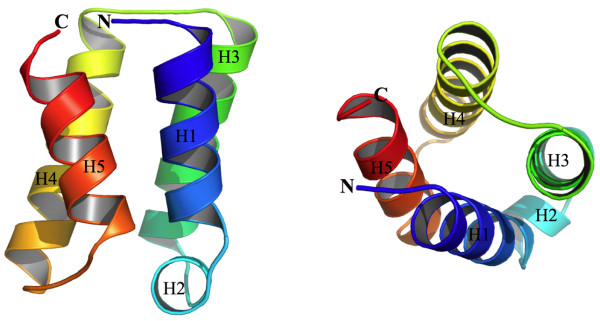
**Structure of **2fu2. The structure of Sp-PIP - PDB ID: 2fu2 - shown as a four helix bundle in two orthogonal views. Both N- and C-termini of proteins are labeled and the protein is colored from blue (N-terminus) to red (C-terminus).

We have expressed the pyogenecin immunity protein from *S. pyogenes *in *Escherichia coli *and purified it to homogeneity. The crystal structure of Sp-PIP was solved by the single wavelength anomalous diffraction (SAD) method using selenomethionine (SeMet)-substituted protein. The model was refined to 2.15 Å with an R-factor of 15.7% and an R-free of 25.4%. The crystal asymmetric unit contains 78 (out of the 102) well ordered residues and 74 water molecules. The first methionine and the 23 C-terminal residues are disordered and cannot be identified in the electron density maps. The structure is high quality; all non-glycine and non-proline residues of the model lie either in the most favorable region (95.9% of residues) or in the additionally allowed region (4.1% of residues) of the Ramachandran plot. Detailed refinement statistics and crystallographic data are shown in Table 1. The crystal structure of Sp-PIP, shown in Figure 1, is a typical anti-parallel four-helix bundle. The four long helices (H1, residues 3-16; H3, 23-41; H4, 43-61; H5, 66-79) pack tightly together around a well-defined hydrophobic core. One additional short helix (H2, residues 17-22) connects helices H1 and H3. All long helices are between 14 and 17 residues in length. Helix-pairs H1/H4 and H2/H3 are parallel to each other and helix-pairs H1/H2 and H3/H4 cross over each other at an angle of approximately 30°. In the structure, the N- and C-termini are very close together (7 Å). Overall, the protein is slightly acidic (calculated pI = 6.06) with two large acidic patches near each end of the bundle and one large positively charged patch near the N- and C-termini.

**Table 1:** Crystallographic results. The crystallographic data and refinement statistics for the structure 2fu2.

Data collection statistics	
Wavelength (Å)	0.9791

Space group	C2

Cell parameters(Å, °)	*a *= 76.09

	*b *= 30.28
	*c *= 35.86
	*β *= 113.26

Resolution (Å)	50-2.15

Total reflections	82,574

Unique reflections	4,042

Completeness (%)	96.3(83.0)

I/sigma	34.3(7.4)

R_merge_(%)	8.1(22.9)

Refinement statistics	

Resolution range (Å)	50-2.15

*R*_cryst_(%)	15.7

*R*_free _(%)	25.4

R.m.s. deviations from ideality	

Bond lengths (Å)	0.026

Angles (°)	1.81

No. of protein atoms	627

No. of solvent atoms	67

Average B-factor	

main-chain atoms (Å^2^)	33.7

Side-chain atoms (Å^2^)	35.4

Protein atoms (Å^2^)	34.6

Solvent atoms (Å^2^)	48.0

### Structural analysis

Structural comparisons, using the DALI server [27], reveal that the structure of Sp-PIP matches a range of proteins all having a four helix bundle topology, with a Z-score of 8.7 or less. In this range of Z-scores, it is not clear whether the matches are due to homology or simply to structural similarity. However, structural homology combined with sequence analysis, enabled us to relate this protein to structures that ranked 2^nd ^(1tdp), 10^th ^(2bl8) and 23^rd ^(2k19) in the list of DALI matches, and the results are shown in Table 2. These homologous proteins are all pediocin-like immunity proteins. Sp-PIP shares a RMSD of 2.4 Å with ImB2, 1tdp, over 75 residues, and, using the structure comparison service SSM at European Bioinformatics Institute http://www.ebi.ac.uk/msd-srv/ssm[28], the RMSD is found to be 2.11 Å.

**Table 2:** DALI comparison of Sp-PIP with other immunity proteins.

Structure with PDB ID	Z-score	RMSD Å	# residues
1tdp -- Carnobacteriocin immunity protein, ImB2	8.3	2.4	75

2bl8 -- Enterocin A immunity protein, EntA-im	7.3	3.0	72

2k19 -- Piscolin 126 immunity protein, PisI	6.9	3.0	75

DALI scores for structures representative of groups A, B and C immunity proteins against Sp-PIP.

### Sequence analysis

Guided by the structure of Sp-PIP, whose sequence had not been assigned to any known protein family, we sought to expand our knowledge of its sequence relatives using the Pfam database [29]. Having identified that it was structurally similar to known members of the Pfam EntA_Immun family (Pfam:PF08951) we carried out an analysis with profile Hidden Markov models (HMMs) to determine whether Sp-PIP should also belong to this family or not. Taking the EntA_Immun family from Pfam release 22.0 as a starting point we carried out iterative searches using the HMMER package (v2.3.2). After multiple rounds of searching of the HMM against the sequence database (UniProt version 12.5) using an E-value threshold of 0.04 along with careful manual inspection of the resulting matches we were able to detect 172 sequences compared to the 19 sequences found in Pfam release 22.0. The family has been updated in the current release of Pfam - 24.0 - and the alignment is now available on the Pfam web-site at: http://pfam.sanger.ac.uk//family/Pf08951.

### Domain analysis

We investigated whether any other protein domains were to be found on any immunity proteins in the EntA_Immun family. Some members of the family do also carry associated domains. One protein contains an N-terminal helix-turn-helix transcriptional regulator (UniProt:Q88Y45), and a group of methionine sulfoxide reductases from *Enterococcus faecium *(UniProt:Q3Y319) and closely related species carry a C-terminal PMSR domain - peptide methionine sulfoxide reductase (Pfam:PF01625) - as well as the N-terminal immunity protein domain.

### Proteome analysis

**Figure 2:**
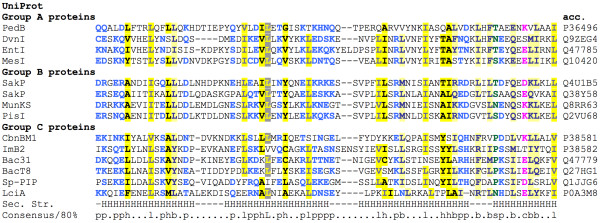
**Alignment of representative immunity proteins**. Multiple sequence alignment of representative immunity proteins from groups A, B and C colored using CHROMA software (Goodstadt and Ponting 2001).

Sp-PIP belongs to a large family of putative bacteriocin immunity proteins. To investigate the possible targets of the presumed immunity protein we carried out an analysis of the proteomes of several strains of *S. pyogenes*, including M1 GAS, MGAS10750, and MGAS8232, to try to identify any complementary bacteriocins and immunity proteins. We searched the proteomes with HMMs of both the known class I, antibacterial18 (Pfam:PF08130), and class II bacteriocins, bacteriocin_II (Pfam:PF01721), bacteriocin_IIc (Pfam:PF10439) as well as with the EntA_Immun families, from the Pfam database (release 23.0). We have identified a small genomic region containing four genes encoding putative bacteriocin proteins of class IIb (Spy_0479, Spy_0480, Spy_0484 and Spy_0486), and have called the proteins Pyogenecin 1, 2, 3, and 4, respectively (UniProt:Q9A139, Q9A138, Q9A137, Q9A136). The organization of this region is shown in Figure 2a. We have also identified two separate gene loci encoding immunity proteins in the *S. pyogenes *genome: Spy_2152 (Sp-PIP) and Spy_1675. Both encoded proteins were found in all the *S. pyogenes *strains examined, and are identified as belonging to the EntA_Immun family (Pfam:PF08951), a family which contains nearly 200 different members from LAB and closely related species.

## Discussion

**Figure 3:**
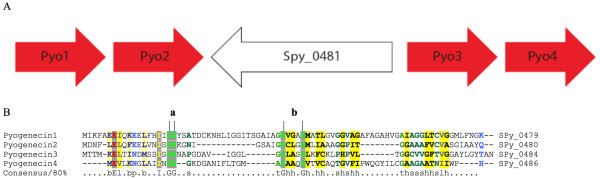
***Streptococcus pyogenes *bacteriocin cluster**. (A) Organization of the bacteriocin locus of *S. pyogenes*, containing four peptides shown by red arrows, Pyogenecin1-4 as Pyo1-4; (B) Multiple sequence alignment of putative bacteriocins using T-Coffee (Notredame *et al*., 2000), colored using CHROMA software (Goodstadt and Ponting 2001). The GG "double-glycine" leader peptide motif is indicated at (a) and the GxxxG helix-interacting motif at (b).

The four-helix bundle structure of Sp-PIP was found to be most closely related to those of a number of pediocin-like IIa immunity proteins. Three subgroups of pediocin-like IIa immunity proteins have been defined on the basis of common sequence motifs and phylogenetic analysis [17], being denoted as groups A, B and C, as shown in Figure 3:

• Group A: EntA-im, the enterocin A immunity protein, [20]; PDB ID:2bl8

• Group B: PisI, the piscicolin 126 immunity protein [22]; PDB ID: 2k19

• Group C: ImB2, the carnobacteriocin B2 immunity protein [19]; PDB ID:1tdp

The structural comparison results suggest that Sp-PIP is most closely related to Imb2, from subgroup C. These results suggest that Sp-PIP is highly likely to function as a bacteriocin immunity protein with similarities to proteins from group C.

Sequence analysis reveals that Sp-PIP belongs to the now expanded Pfam family of immunity proteins EntA_Immun that also includes pediocin-like immunity proteins from groups A, B and C as detailed above, thus confirming its evolutionary link to known bacteriocin immunity proteins. A search for other immunity proteins of a similar nature in the *S. pyogenes *genome identified another gene locus encoding a protein matching the EntA_Immun family. Both of these loci were seen in all strains examined. Unlike the case in many other species, in *S. pyogenes *these bacteriocin immunity proteins were not found closely linked to their bacteriocins, a condition that might be explained by the remnants of a transposon identified between them.

Functions for proteins may often be inferred through consideration of the combinations of the functional domains found on them. A number of domain-pairings were identified on some of the immunity family members, such as an N-terminal helix-turn-helix transcriptional regulator on one protein, which might be suggestive of immunity proteins acting as transcriptional regulators or of interacting with transcription regulators and modulating their function. A group of methionine sulfoxide reductases are found to carry both a C-terminal PMSR domain and the immunity domain. The PMSR domain is involved in reversing protein inactivation by oxidation of methionine residues. However, it is not clear what, if any, functional significance these domain-pairings might have.

The search for potential bacteriocin targets for this putative immunity protein took in classes I, II and III. Our analyses identified that *S. pyogenes *contains a class I lantibiotic system composed of one unique lantibiotic type A sequence called *srtA *and just one unique immunity protein *srtI*. These two genes are closely associated on the genome, but are separated from the proteins of the class II system described below as well as from the presumed immunity proteins. This suggests that the Sp-PIP is unlikely to act as an immunity protein for class I bacteriocins. Class II bacteriocins are subdivided into classes IIa, IIb, IIc and IId. We did not find any bacteriocins of class IIa encoded by the *S. pyogenes *genome, which would have the well-conserved N-terminal YGNGVxCxxxC motif [30,31]. Searches were not made for bacteriocins of types IIc or IId since the immunity proteins complementary for these two groups identified so far, gassericin A [32] and circularin A, [33] do not share sequence or size similarity with those of the pediocin-like immunity proteins. The four putative class IIb proteins we identified from a short genomic region, and named pyogenecins 1-4, are found as two tandem pairs of genes separated by a gene in the antisense orientation that encodes a transposase pseudogene. The presence of this pseudogene suggests that this region might once have been part of a mobile genetic element. Finding bacteriocins in pairs is characteristic of the class IIb bacteriocins [34]. As with other class IIb bacteriocins, these pyogenecins have a conserved GG leader peptide, as well as the conserved GXXXG motif necessary for helix-helix interaction between the two proteins, as illustrated in Figure 2b. All four putative pyogenecins are found to fall into the Pfam Bacteriocin_IIc family (Pfam:PF10439), a family of bacteriocins secreted by streptococcal and other LAB species.

Based on the sequence similarity to other two-peptide bacteriocins, the genomic arrangement of the genes found, and the identification of two immunity proteins, we hypothesize that the putative pyogenecins 1 and 2 associate to form one active bacteriocin and pyogenecins 3 and 4 associate to form a second active bacteriocin possibly targeting a different bacterial species. These peptides need now to be synthesized to allow testing of their biological activity in a suitable bacteriocin-assay. Given that we find two immunity proteins we suggest that each one is specific for one of the putative bacteriocin pairs, Pyogenecin1/Pyogenecin2 and Pyogenecin3/Pyogenecin4.

There is also the possibility that one or both of these immunity proteins might be an orphan immunity protein [17], so-called because no cognate bacteriocin in the genome has been identified; and it has been pointed out that most other immunity proteins so far determined for IIa two-peptide bacteriocins are small trans-membrane proteins closely coupled transcriptionally with their bacteriocins [9]. However, it would appear that the sequences of only a handful of two-peptide immunity proteins have been deduced and neither are there defined structures for any of these nor are most of them more than putative immunity proteins, the designation being based only on the presence of predicted transmembrane helices [35-37], so comparison with the system we propose here is difficult. The exact method by which immunity proteins protect their host cell or interact with the bacteriocins is also unclear, but it may be that the orphan immunity proteins do confer resistance to other bacteriocins in addition to their cognate bacteriocin [17,9]. It is possible of course that many more two-peptide-like systems remain still to be characterized, as there are a large number of additional LAB peptides in the Pfam family Bacteriocin_IIc PF10439 which are "pairs" in the sense that they are expressed from closely adjacent genes and for which a cognate immunity protein has not yet been identified.

## Conclusions

The structure of the putative immunity protein Sp-PIP protective against potential class IIb two-peptide bacteriocins could be the first structural representative of this class. A combined structural, genomic and proteomic analyses has allowed the identification and *in silico *characterization of a new putative immunity protein from *S. pyogenes*. Structurally similar proteins seem to provide immunity protection for single peptide IIa class bacteriocins. Further biological and biochemical studies are needed to verify the antibacterial activity of the putative pyogenecins and to determine the degree of resistance and cross-resistance provided by the proposed immunity protein.

## Methods

### Protein cloning, expression and purification

The ORF from the Spy_2152 locus was amplified from genomic *S. pyogenes *M1 DNA with *KOD *DNA polymerase using conditions and reagents provided by the vendor (Novagen, Madison, WI). The gene was cloned into the pMCSG7 vector using a modified ligation-independent cloning protocol [38] and over-expressed in *E. coli *BL21 (DE3) - Gold (Stratagene) harboring an extra plasmid encoding three rare tRNAs (AGG and AGA for Arg, ATA for Ile). The pMCSG7 vector bearing a tobacco etch virus (TEV) protease cleavage site creates a construct with a cleavable His_6_-tag fused onto the N-terminus of the target protein and adds three artificial residues (Ser-Asn-Ala) on that end. The cells were grown using SeMet-containing enriched M9 medium and conditions known to inhibit methionine biosynthesis. The cells were grown at 37°C to an OD_600 _of ~0.6 and protein expression induced with 1 mM isopropyl-1-thio-β-D-galactopyranoside (IPTG). After induction, the cells were grown overnight with shaking at 20°C. The harvested cells were re-suspended in lysis buffer buffer (50 mM HEPES pH 8.0, 500 mM NaCl, 10 mM imidazole, 10 mM β-mercaptoethanol, and 5% v/v glycerol) in the presence of lysozyme (1 mg/mL) and of protease inhibitor cocktail (100 μL per 2 g of wet cells), and then kept on ice for 20 min before sonication. Then the cells were lysed using sonication. The lysate was clarified by centrifugation at 30,000 × g (RC5C-Plus centrifuge, Sorval) for 20 min, followed by filtration through 0.45 μm and 0.22 μm in-line filters (Gelman). The lysate was then applied to a 5 mL HiTrap Ni-NTA column on the AKTAxpress (GE Health Systems). His_6_-tagged protein was eluted using buffer containing a higher concentration of imidazole (500 mM NaCl, 5% glycerol, 50 mM HEPES, pH 8.0, 250 mM imidazole, 10 mM 2-mercaptoethanol), and the His_6_-tag was cleaved from the protein by treatment with recombinant His_6_-tagged TEV protease. A second Ni-NTA affinity chromatography was performed manually to remove the His_6_-tag and His_6_-tagged TEV protease. A total of 90 mg of protein was purified for finding crystallization conditions. Then the protein was dialyzed in 20 mM Tris-HCl pH 7.1, 50 mM NaCl, 2 mM dithiothreitol (DTT) and concentrated to 110 mg/mL using a Centricon Plus-20 Centrifugal Concentrator (Millipore). The standard purification protocol has been thoroughly described previously [39].

### Protein crystallization

The initial crystallization conditions were searched using the sitting drops vapor diffusion method at 18°C with the help of the Mosquito crystallization workstation (TTP Labtech) with Index (Hampton Research) and Wizard I and II (Emerald Biostructures) crystallization screens. Crystals suitable for X-ray experiment were obtained at the initial stage of crystal screening, the crystallization condition number 23 of Wizard II within several days. Crystals were flash-frozen in liquid nitrogen with crystallization solution containing 25% (v/v) glycerol as cryoprotectant prior to data collection.

### Data collection and structure determination

Anomalous diffraction data were collected at the selenium peak from crystals of a SeMet-substituted protein. The data sets were collected on an ADSC quantum Q315 charge-coupled device-detector at 100 K on the Structural Biology Center beamline 19ID at the Advanced Photon Source, Argonne National Laboratory. The space group was found to be C2, with cell parameters of *a *= 76.09 Å, *b *= 30.28 Å, *c *= 35.86 Å, α = 90.00°, β = 113.26°, and γ = 90.00°. The diffraction data were processed using the HKL3000 suite of programs [40].

All procedures for single wavelength anomalous dispersion (SAD), phasing, phase improvement by density modification, and initial protein model building were done using the structure module of the HKL3000 software package. The mean figure of merit of the phase-set was 0.207 for 50-2.25 Å data and improved to 0.542 after density modification (DM). The autotracing Arp/wArp module [41] in HKL3000 built 77 out of 102 residues with fitted sequence. The initial model was rebuilt with the program COOT [42] using electron density maps based on DM-phased reflection file. After each cycle of rebuilding, the model was refined using REFMAC5 [43] from the CCP4 suite with TLS refinement. The stereochemistry of the structure was checked with PROCHECK [44]. Atomic coordinates and experimental structure factors of Sp-PIP have been deposited with the PDB database and are accessible under the code 2fu2.

## Authors' contributions

CC, LV, MC performed the crystallography part of the research; AB, PC and RF performed the genome sequence searches; PC, AB, ZO, WM and AJ analyzed data; AJ designed the research; PC, AB and AJ wrote the manuscript. All authors read and approved the final manuscript.

## References

[B1] Drider D Fimland G "The continuing story of class IIa bacteriocins" Microbiol Mol Biol Rev 2006 70 2 564 82 10.1128/MMBR.00016-05 16760314 PMC1489543

[B2] Cotter PD Hill C "Bacteriocins: developing innate immunity for food." Nat Rev Microbiol 2005 3 10 777 88 10.1038/nrmicro1273 16205711

[B3] Ennahar S Sashihara T "Class IIa bacteriocins: biosynthesis, structure and activity." FEMS Microbiol Rev 2000 24 1 85 106 10.1111/j.1574-6976.2000.tb00534.x 10640600

[B4] Galvez A Abriouel H "Bacteriocin-based strategies for food biopreservation." Int J Food Microbiol 2007 120 1-2 51 70 10.1016/j.ijfoodmicro.2007.06.001 17614151

[B5] Tichaczek PS Vogel RF "Cloning and sequencing of curA encoding curvacin A, the bacteriocin produced by Lactobacillus curvatus LTH1174." Arch Microbiol 1993 160 4 279 83 10.1007/BF00292077 7694558

[B6] Uteng M Hauge HH "Three-dimensional structure in lipid micelles of the pediocin-like antimicrobial peptide sakacin P and a sakacin P variant that is structurally stabilized by an inserted C-terminal disulfide bridge." Biochemistry 2003 42 39 11417 26 10.1021/bi034572i 14516192

[B7] Haugen HS Fimland G "Three-dimensional structure in lipid micelles of the pediocin-like antimicrobial peptide curvacin A." Biochemistry 2005 44 49 16149 57 10.1021/bi051215u 16331975

[B8] Fimland N Rogne P "Three-dimensional structure of the two peptides that constitute the two-peptide bacteriocin plantaricin EF." Biochim Biophys Acta 2008 1784 11 1711 9 18555030 10.1016/j.bbapap.2008.05.003

[B9] Oppegard C Rogne P "The two-peptide class II bacteriocins: structure, production, and mode of action." J Mol Microbiol Biotechnol 2007 13 4 210 9 10.1159/000104750 17827971

[B10] Rogne P Fimland G "Three-dimensional structure of the two peptides that constitute the two-peptide bacteriocin lactococcin G." Biochim Biophys Acta 2008 1784 3 543 54 18187052 10.1016/j.bbapap.2007.12.002

[B11] Leer RJ Vossen JM van der "Genetic analysis of acidocin B, a novel bacteriocin produced by Lactobacillus acidophilus." Microbiology 1995 141 Pt 7 1629 35 10.1099/13500872-141-7-1629 7551031

[B12] Kemperman R Kuipers A "Identification and characterization of two novel clostridial bacteriocins, circularin A and closticin 574." Appl Environ Microbiol 2003 69 3 1589 97 10.1128/AEM.69.3.1589-1597.2003 12620847 PMC150056

[B13] Wirawan RE Swanson KM "Uberolysin: a novel cyclic bacteriocin produced by Streptococcus uberis." Microbiology 2007 153 Pt 5 1619 30 10.1099/mic.0.2006/005967-0 17464077

[B14] Maqueda M Sanchez-Hidalgo M "Genetic features of circular bacteriocins produced by Gram-positive bacteria." FEMS Microbiol Rev 2008 32 1 2 22 10.1111/j.1574-6976.2007.00087.x 18034824

[B15] Axelsson L Holck A "The genes involved in production of and immunity to sakacin A, a bacteriocin from Lactobacillus sake Lb706." J Bacteriol 1995 177 8 2125 37 7721704 10.1128/jb.177.8.2125-2137.1995 PMC176857

[B16] Quadri LE Kleerebezem M "Characterization of a locus from Carnobacterium piscicola LV17B involved in bacteriocin production and immunity: evidence for global inducer-mediated transcriptional regulation." J Bacteriol 1997 179 19 6163 71 9324267 10.1128/jb.179.19.6163-6171.1997 PMC179523

[B17] Fimland G Eijsink VG "Comparative studies of immunity proteins of pediocin-like bacteriocins." Microbiology 2002 148 Pt 11 3661 70 12427956 10.1099/00221287-148-11-3661

[B18] Johnsen L Fimland G "The C-terminal domain of pediocin-like antimicrobial peptides (class IIa bacteriocins) is involved in specific recognition of the C-terminal part of cognate immunity proteins and in determining the antimicrobial spectrum." J Biol Chem 2005 280 10 9243 50 10.1074/jbc.M412712200 15611086

[B19] Sprules T Kawulka KE "NMR solution structure of ImB2, a protein conferring immunity to antimicrobial activity of the type IIa bacteriocin, carnobacteriocin B2." Biochemistry 2004 43 37 11740 9 10.1021/bi048854+ 15362858

[B20] Johnsen L Dalhus B "1.6-Angstroms crystal structure of EntA-im. A bacterial immunity protein conferring immunity to the antimicrobial activity of the pediocin-like bacteriocin enterocin A." J Biol Chem 2005 280 19 19045 50 10.1074/jbc.M501386200 15753083

[B21] Kim IK Kim MK "High resolution crystal structure of PedB: a structural basis for the classification of pediocin-like immunity proteins." BMC Struct Biol 2007 7 35 10.1186/1472-6807-7-35 17537233 PMC1904221

[B22] Martin-Visscher LA Sprules T "Nuclear magnetic resonance solution structure of PisI, a group B immunity protein that provides protection against the type IIa bacteriocin piscicolin 126, PisA." Biochemistry 2008 47 24 6427 36 10.1021/bi8004076 18500825

[B23] Jeon HJ Noda M "Crystal structure and mutagenic analysis of a bacteriocin immunity protein, Mun-im." Biochem Biophys Res Commun 2009 378 3 574 8 10.1016/j.bbrc.2008.11.093 19061861

[B24] Cunningham MW "Pathogenesis of group A streptococcal infections." Clin Microbiol Rev 2000 13 3 470 511 10.1128/CMR.13.3.470-511.2000 10885988 PMC88944

[B25] Robson CL Wescombe PA "Isolation and partial characterization of the Streptococcus mutans type AII lantibiotic mutacin K8." Microbiology 2007 153 Pt 5 1631 41 10.1099/mic.0.2006/003756-0 17464078

[B26] Lee SW Mitchell DA "Discovery of a widely distributed toxin biosynthetic gene cluster." Proc Natl Acad Sci USA 2008 105 15 5879 84 10.1073/pnas.0801338105 18375757 PMC2311365

[B27] Holm L Park J "DaliLite workbench for protein structure comparison." Bioinformatics 2000 16 6 566 7 10.1093/bioinformatics/16.6.566 10980157

[B28] Krissinel E Henrick K "Detection of protein assemblies in crystals." Computational Life Sciences: Proceedings of the First International Symposium, Complife 2005 Berlin Heidelberg: Springer-Verlag 163 174

[B29] Finn RD Tate J "The Pfam protein families database." Nucleic Acids Res 2008 36 Database D281 8 18039703 10.1093/nar/gkm960 PMC2238907

[B30] Cintas LM Casaus P "Biochemical and genetic characterization of enterocin P, a novel sec-dependent bacteriocin from Enterococcus faecium P13 with a broad antimicrobial spectrum." Appl Environ Microbiol 1997 63 11 4321 30 9361419 10.1128/aem.63.11.4321-4330.1997 PMC168752

[B31] Bennik MH Vanloo B "A novel bacteriocin with a YGNGV motif from vegetable-associated Enterococcus mundtii: full characterization and interaction with target organisms." Biochim Biophys Acta 1998 1373 1 47 58 10.1016/S0005-2736(98)00086-8 9733915

[B32] Kawai Y Kusnadi J "DNA sequencing and homologous expression of a small peptide conferring immunity to gassericin A, a circular bacteriocin produced by Lactobacillus gasseri LA39." Appl Environ Microbiol 2009 75 5 1324 30 10.1128/AEM.02485-08 19114506 PMC2648179

[B33] Kemperman R Jonker M "Functional analysis of the gene cluster involved in production of the bacteriocin circularin A by Clostridium beijerinckii ATCC 25752." Appl Environ Microbiol 2003 69 10 5839 48 10.1128/AEM.69.10.5839-5848.2003 14532033 PMC201212

[B34] Martinez B Fernandez M "Synthesis of lactococcin 972, a bacteriocin produced by Lactococcus lactis IPLA 972, depends on the expression of a plasmid-encoded bicistronic operon." Microbiology 1999 145 Pt 11 3155 61 10589723 10.1099/00221287-145-11-3155

[B35] Marciset O Jeronimus-Stratingh MC "Thermophilin 13, a nontypical antilisterial poration complex bacteriocin, that functions without a receptor." J Biol Chem 1997 272 22 14277 84 10.1074/jbc.272.22.14277 9162062

[B36] McCormick JK Poon A "Genetic characterization and heterologous expression of brochocin-C, an antibotulinal, two-peptide bacteriocin produced by Brochothrix campestris ATCC 43754." Appl Environ Microbiol 1998 64 12 4757 66 9835559 10.1128/aem.64.12.4757-4766.1998 PMC90919

[B37] Stephens SK Floriano B "Molecular analysis of the locus responsible for production of plantaricin S, a two-peptide bacteriocin produced by Lactobacillus plantarum LPCO10." Appl Environ Microbiol 1998 64 5 1871 7 9572965 10.1128/aem.64.5.1871-1877.1998 PMC106244

[B38] Dieckman L Gu M "High-throughput methods for gene cloning and expression." Protein Expr Purif 2002 25 1 1 7 10.1006/prep.2001.1602 12071692

[B39] Kim Y Dementieva I "Automation of protein purification for structural genomics." J Struct Funct Genomics 2004 5 1-2 111 8 10.1023/B:JSFG.0000029206.07778.fc 15263850 PMC2778303

[B40] Minor W Cymborowski M "HKL-3000: the integration of data reduction and structure solution--from diffraction images to an initial model in minutes." Acta Crystallogr D Biol Crystallogr 2006 62 Pt 8 859 66 10.1107/S0907444906019949 16855301

[B41] Perrakis A Morris R "Automated protein model building combined with iterative structure refinement." Nat Struct Biol 1999 6 5 458 63 10.1038/8263 10331874

[B42] Emsley P Cowtan K "Coot: Model-Building Tools for Molecular Graphics." Acta Crystallographica Section D - Biological Crystallography 2004 60 Pt 12 Pt 1 2126 32 10.1107/S0907444904019158 15572765

[B43] Murshudov GN Vagin AA "Refinement of macromolecular structures by the maximum-likelihood method." Acta Crystallogr D Biol Crystallogr 1997 53 Pt 3 240 55 10.1107/S0907444996012255 15299926

[B44] Laskowski RA MacArthur MW "PROCHECK: a program to check the stereochemical quality of protein structures." J Apple Cryst 1993 26 283 291 10.1107/S0021889892009944

